# State Medical Assocsation News

**Published:** 1904-10

**Authors:** 


### State Medical Assocsation News.

The Fifth District Medical Society, composed of the counties of Edwards, Kerr, Bandera, Gillespie, Kendall, Uvalde, Zavala, Kinney, Medina, Maverick, Dimmit, La Salle, Karnes, Comal and Val Verde, have begun the publication of a quarterly pamphlet called *"The Fifth District Medical Bulletin."* We have read the first number, August, 1904. It it not a "medical journal," and is sent to members of the society free. There is no subscription list or exchange list. It is to be devoted to matters of interest to the members. We reproduce in this issue a suggestion as to how county medical societies should be conducted. The establishment of such a publication is most commendable, and the first issue is very creditable to the society and the editors, Drs. J. S. Lankford and J. V. Spring. The Fifth District (twenty counties) makes an excellent showing. In the thirteen counties organized up to April 26, 1904, of 201 physicians, 195 (97 per cent) are enrolled as active members. The unorganized counties are Atascosa, Frio, Zavala, Dimmit and La Salle; all sparsely settled.

